# FOXO1 Regulates Bacteria-Induced Neutrophil Activity

**DOI:** 10.3389/fimmu.2017.01088

**Published:** 2017-09-04

**Authors:** Guangyu Dong, Liang Song, Chen Tian, Yu Wang, Fang Miao, Jiabao Zheng, Chanyi Lu, Sarah Alsadun, Dana T. Graves

**Affiliations:** ^1^Department of Periodontics, School of Dental Medicine, University of Pennsylvania, Philadelphia, PA, United States; ^2^Department of Stomatology, The Fifth People’s Hospital of Shanghai, Fudan University, Shanghai, China; ^3^Department of Implantology, Stomatology Hospital, School of Medicine, Zhejiang University, Hangzhou, China; ^4^Shanxi Province People’s Hospital, Taiyuan, China; ^5^State Key Laboratory of Oral Diseases, West China Hospital of Stomatology, Sichuan University, Chengdu, China

**Keywords:** bacteria, forkhead, FOXO, FOXO1 overexpression, host response, infection, inflammation PMN, TLR2

## Abstract

Neutrophils play an essential role in the innate immune response to microbial infection and are particularly important in clearing bacterial infection. We investigated the role of the transcription factor FOXO1 in the response of neutrophils to bacterial challenge with *Porphyromonas gingivalis in vivo* and *in vitro*. In these experiments, the effect of lineage-specific FOXO1 deletion in LyzM.Cre^+^FOXO1^L/L^ mice was compared with matched littermate controls. FOXO1 deletion negatively affected several critical aspects of neutrophil function *in vivo* including mobilization of neutrophils from the bone marrow (BM) to the vasculature, recruitment of neutrophils to sites of bacterial inoculation, and clearance of bacteria. *In vitro* FOXO1 regulated neutrophil chemotaxis and bacterial killing. Moreover, bacteria-induced expression of CXCR2 and CD11b, which are essential for several aspects of neutrophil function, was dependent on FOXO1 *in vivo* and *in vitro*. Furthermore, FOXO1 directly interacted with the promoter regions of CXCR2 and CD11b. Bacteria-induced nuclear localization of FOXO1 was dependent upon toll-like receptor (TLR) 2 and/or TLR4 and was significantly reduced by inhibitors of reactive oxygen species (ROS and nitric oxide synthase) and deacetylases (Sirt1 and histone deacetylases). These studies show for the first time that FOXO1 activation by bacterial challenge is needed to mobilize neutrophils to transit from the BM to peripheral tissues in response to infection as well as for bacterial clearance *in vivo*. Moreover, FOXO1 regulates neutrophil function that facilitates chemotaxis, phagocytosis, and bacterial killing.

## Introduction

Neutrophils are the first line of defense against invading pathogens (1). Bacteria induce neutrophil recruitment and mobilization. Neutrophils are recruited to sites of injury or infection early in the inflammatory process. After mobilization from the bone marrow (BM), neutrophils are rapidly recruited to the in infected peripheral tissues (2). This process is an important early step in controlling tissue infections (2). In mice, the chemokines CXCL1/CXCL2 stimulate recruitment of neutrophils *via* CXCR2 (3, 4). Neutrophils lacking CXCR2 are preferentially retained in the BM and have deficient recruitment of neutrophils following infection (5, 6). Thus, CXCL1-CXCR2-mediated neutrophil recruitment plays a critical role in protecting the host from bacterial infection (7–9).

Phagocytosis is a critical antimicrobial function of neutrophils that is needed to remove bacteria (10, 11). Complement factors C3b and C3bi opsonize bacteria, which in turn are phagocytized by neutrophils that carry the surface receptor CD11b/CD18 integrin, also known as complement receptor 3 (12). After bacteria are phagocytosed, they are killed and lysed in lysosomes (13). Bacteria stimulate neutrophils through pattern recognition receptors including toll-like receptors (TLRs). TLR2 and TLR4 are membrane receptors that recognize pathogen-associated molecular patterns (14). After interacting with bacteria, TLR2 and TLR4 stimulate secretion of cytokines (15). TLR2/4 has been shown to induce a number of transcription factors that induce antimicrobial activity in neutrophils. A transcription factor that has gained attention recently for its role in dendritic and lymphocyte function is FOXO1. We have recently shown that deletion of FOXO1 reduces dendritic cell function and impairs the ability of dendritic cells to activate the adaptive immune response (16). Previous results demonstrate that FOXO1 mediates LPS-induced cytokine expression in these cells (17). FOXO1 is needed for dendritic cell migration and homing to lymph nodes by regulating CCR7 and ICAM-1 expression (16). FOXO1 promotes lymphocyte homeostasis by regulating CCR7 expression *via* binding to the promoter region of CCR7 in T cells (18, 19). FOXO1 induces monocyte/macrophage activation and differentiation but does not affect CD11b expression (20).

Although it is well recognized that neutrophils are critical in the initial response to bacterial challenge and bacterial clearance, the mechanisms that control this response have not been fully explored. It is appreciated that TLRs play a key role in activation of neutrophils. However, the range of transcription factors that are triggered by bacteria-induced TLR signaling and their downstream gene targets have not been fully explored. The activation and function of the transcription factor FOXO1 in the neutrophil response to bacteria is unknown. To investigate the role of FOXO1 in neutrophil function, we examined mice with lineage specific deletion of FOXO1. The results indicate that FOXO1 activity is stimulated in neutrophils, that FOXO1 regulates CD11b and CXCR2 and that FOXO1 mediates phagocytosis and bacterial killing, which are important for bacterial clearance. Furthermore, FOXO1 contributes to mobilizing neutrophil movement from a BM compartment to peripheral tissue.

## Materials and Methods

### Mice

Mice that express Cre recombinase under control of the lysozyme M promoter (LyzM^+^.Cre) were purchased from The Jackson Laboratory (Bar Harbor, ME, USA). FOXO1^L/L^ mice were generously provided by Dr. Ronald DePinho (University of Texas MD Anderson Cancer Center, Houston, TX, USA) (21). FOXO1^L/L^ mice were bred with LyzM.Cre mice to generate experimental mice (LyzM.Cre^+^FOXO1^L/L^) and the control littermates (LyzM.Cre^−^FOXO1^L/L^) as described (20). Genotypes were determined by PCR using primers specific for LyzM.Cre (5′-ATCCGAAAAGAAAACGTTGA-3′ and 5′-ATCCAGGTTACGGATATAGT-3′) and specific for FOXO1 (5′-GCTTAGAGCAGAGATGTTCTCACATT-3′, 5′-CCAGAGTCTTTGTATCAG GCAAATAA-3′, and 5′-CAAGTCCATTAATTCAGCACATTG A-3′). All procedures were approved by the Institutional Animal Care and Use Committee of the University of Pennsylvania.

### Bacterial Strains and Animal Injection

Broth-grown *Porphyromonas gingivalis* (ATCC, #33277) in logarithmic growth phase was collected and washed three times with phosphate-buffered saline (PBS). Bacteria were then resuspended and counted with a standard CFU curve as previously described (22). Mice were challenged by injection of lightly fixed or live *P. gingivalis* (ATCC, #33277) or sham injection with vehicle alone (PBS) into the scalp connective tissue as described (23–25) and euthanized at indicated time points after the injection (26). Neutrophils were isolated from the vasculature, BM, and scalp connective tissues and assessed by flow cytometry after incubation with specific antibodies or control IgG as previously described (27). Neutrophil mobilization was calculated as described (28).

### Neutrophil Isolation and Cell Culture

Primary mouse neutrophils were isolated from the BM (27). Briefly, BM cells from experimental mice (LyzM.Cre^+^FOXO1^L/L^) and the control littermates (LyzM.Cre^−^FOXO1^L/L^) were suspended in PBS without calcium and magnesium, placed over Histopaque^®^ 1119 and 1077 (Sigma Chemicals Ltd.) and centrifuged at 2,000 rpm, 25°C for 30 min. The neutrophil layer was collected and washed twice in PBS. Neutrophil purity was routinely >95%, as determined by flow cytometry after staining for Ly6G, F4/80, and CD3 (27). Primary human neutrophils were isolated from human peripheral blood (PBL) of healthy donors obtained from the Human Immunology Core at University of Pennsylvania following the procedure in Ref. (27). Neutrophil purity (routinely >95%) was determined by Wright–Giemsa staining (27).

The human promyelocytic leukemia HL-60 cells (ATCC CCL-240) were cultured at 37°C in 5% CO_2_ in RPMI 1640 supplemented with 2 mM l-glutamine, 25 mM HEPES, and 10% heat-inactivated fetal bovine serum (MilliporeSigma, St. Louis, MO, USA). HL-60 cells were incubated with 1.3% DMSO (MilliporeSigma) for 4 days (29) and their differentiation into neutrophils (referred to as HL-60 neutrophils) was monitored by flow cytometry analysis by CD11b expression using antihuman CD11b mAb (BD Pharmingen) (27).

### Neutrophil Migration

Chemotaxis was measured in primary mouse BM neutrophils with transwell chambers (polycarbonate filter, 5-µm pore size, Corning) with or without CXCL1 (Peprotech) for 2 h at 37°C in the bottom chamber. Neutrophils that migrated to the bottom side of the filter were counted by DAPI staining and fluorescence microscopy.

### Neutrophil Phagocytosis, Clearance and Bacterial Killing

Bacterial phagocytosis was performed as described (30) with modification. Briefly, bacteria were labeled with *CFSE* (#65-0850-84, carboxyfluorescein succinimidyl ester, Thermo Fisher Scientific, Waltham, MA, USA) (31) and incubated with neutrophils at multiplicity of infection (MOI) 1:10 (cell: bacteria) for 1 h. The neutrophils were fixed and stained with Alexa 647-labeled wheat germ agglutinin (WGA) (W32466, Thermo Fisher Scientific) to delineate the cell surface (red) and internalized bacteria (green) then visualized by fluorescent microscopy with deconvolution. Internalized bacteria were considered as those within WGA-decorated plasma membranes (27). Neutrophil-associated bacteria were determined by colocalizing CFSE-labeled *P. gingivalis* and Alexa 647-labeled WGA stained neutrophils. Internalized bacteria and neutrophils with associated bacteria were counted under fluorescent microcopy (27). Bacterial clearance *in vivo* was determined by inoculating *P. gingivalis* (1 × 10^7^ bacteria/injection) into the scalp connective tissue of experimental LyzM.Cre^+^FOXO1^L/L^ and LyzM.Cre^−^FOXO1^L/L^ control littermates, a well-characterized experimental model (23–26). The scalp soft tissue was harvested, mechanically processed by medimachine (BD Biosciences, San Jose, CA, USA), lysed, and divided into aliquots with multiple dilutions that were used for numeration of recovered CFU (colonies were counted after anaerobic culture on blood agar plates). Data are presented as total number of bacteria recovered per mouse (27). To assess bacterial killing *in vitro* mouse neutrophils were cocultured with *P. gingivalis* (MOI = 1:1) (cell: bacteria) at 37°C and 5% CO_2_ for 2 h. The neutrophils were lysed and viable CFU were enumerated after anaerobic culture on blood agar plates. The neutrophil killing index was calculated according to the formula: [(CFU in the absence of neutrophils − CFU in the presence of neutrophils)/CFU in the absence of neutrophils] × 100 (27).

### Treatment by Inhibitors

The inhibitors for reactive oxygen species (ROS, NAC, N-acetyl-l-cysteine, 5 mM) (32), nitric oxide synthase (NOS, l-NAME, NG-nitro-l-arginine methyl ester, 5 mM) (33), Sirt1 (Sirtinol, 10 µM), histone deacetylases (HDAC, Trichostatin A, TSA, 2 µM), TLR4 (TAK242, 1 µg/mL), and DMSO were obtained from MilliporeSigma (Billerica, MA, USA) or Cayman Chemical (Ann Arbor, MI, USA). TLR2 blocking antibody (10 μg/mL) and matched mouse IgG2a (10 µg/mL) were obtained from Santa Cruz Biotechnology (Dallas, TX, USA). HL-60 neutrophils were incubated with inhibitors or antibody compared to vehicle or matched control IgG for 1 h before *P. gingivalis* challenge and during incubation with bacteria. After 16 h, cells were fixed and examined by immunofluorescence with antibody to FOXO1 compared to matched control IgG. FOXO1 nuclear localization was assessed by FOXO1 colocalization with DAPI nuclear stain by image analysis with NIS-Elements software (Nikon, Melville, NY, USA).

### Immunofluorescence Analysis

HL-60 neutrophils were incubated with *P. gingivalis* in 96-well plates for 12 h at 37°C. Neutrophils were fixed in 3.7% formaldehyde for 10 min, permeabilized in 0.5% Triton X-100 for 5 min, blocked in 2% BSA, and stained with primary antibody and appropriate isotype-matched negative control antibody. This was followed by incubation with biotinylated secondary antibody and ABC reagent and visualized by incubation with Alexa Fluor 546-conjugated streptavidin (#S11225, Thermo Fisher Scientific) with DAPI counterstain (#62248, Thermo Fisher Scientific). Images were captured at a magnification of 200× with a fluorescence microscope (Nikon) with the same exposure time for experimental and negative control groups. The capture time was set so that control antibody images were negative. Image analysis was performed using NIS Elements AR image analysis software. The percentage of immunofluorescence positive cells or mean fluorescence intensity (MFI) was measured.

### Transient Transfection and Quantitative Real-time PCR

Neutrophils were transfected with a plasmid containing constitutively active FOXO1, FOXO1-AAA (referred to as FOXO1), or pcDNA empty vector as we have described (34) by electroporation with Amaxa Nucleofector Transfection Device (Lonza, Basel, Switzerland) or Neon Transfection System (Thermo Fisher Scientific) following the manufacturer’s instructions. Cells were then stimulated with *P. gingivalis* as MOI 1:10 overnight. Total RNA was extracted from neutrophils and gene expression was then measured by quantitative real-time PCR with primers (IDT, Coralville, IA, USA) designed using the Universal Probe Library Assay Design Center (Roche Applied Science, Indianapolis, IN, USA) and labeled probes (Roche Applied Science). Each value was normalized to ribosomal protein L32 and represents the mean of three independent experiments.

Human primary neutrophils were transfected with FOXO1 siRNA as described (27). Briefly, ON-TARGET plus SMART pool siRNAs specific for FOXO1 and control scrambled non-targeting control pool siRNA were obtained from GE Healthcare Lifesciences (Pittsburgh, PA, USA) and transfection was performed using lipofectamine 3000 Transfection Reagent (L3000008, Thermo Fisher Scientific) according to the manufacturer’s instructions.

### Western Blot

Neutrophils were lysed with lysis buffer (sc-24948, Santa Cruz Biotechnology) containing protease inhibitor cocktail and phosphatase inhibitor cocktail. Protein concentration was measured using a protein assay with BSA as a standard (#26149, Thermo Fisher Scientific). The 30–60 µg cell lysate was resolved in 4–20% SDS-PAGE (#4561084, Bio-Rad Laboratories) and transferred onto PVDF membrane (#88518, Thermo Fisher Scientific). The membranes were incubated with primary antibodies against FOXO1 (#2880S, 1:500, rabbit mAb, Cell Signaling Technology) and β-actin (A5316, 1:1,000, MilliporeSigma) after blocking with 5% milk. The samples were then incubated with horseradish peroxidase-labeled donkey antirabbit IgG (NA934, 1:5,000, GE Healthcare) or antimouse IgG (HAF018, 1:5,000, R&D), and immunoreactive bands were detected with ECL Western blotting reagents (#32209, Thermo Fisher Scientific).

### Chromatin Immunoprecipitation (ChIP) Assays

Chromatin immunoprecipitation assays were performed using a ChIP-IT Kit (#53035, Active Motif, Carlsbad, CA, USA). Cells were fixed in 1% formaldehyde, DNA sheared enzymatically and immunoprecipitated with anti-FOXO1 or matched control antibody and captured with magnetic protein G beads. The precipitated DNA was then amplified by real-time SYBR green real-time PCR using primers for CD11b and CXCR2.

### Apoptosis *In Vitro*

Apoptosis were measured by flow cytometry with Annexin V FITC apoptosis detection kit (#88-8005, Thermo Fisher Scientific) according to the manufacturer’s instructions. In brief, neutrophils were coculture with *P. gingivalis* for overnight and assessed by flow cytometry after stained with Annexin V. Data were analyzed by Flow Jo software.

### Statistical Analysis

Experiments were carried out a minimum of two to three times with similar results. Statistical significance was determined by *t*-test or ANOVA with Turkey’s *post hoc* test at *P* < 0.05.

## Results

### FOXO1 Deletion Impairs Bacteria-Induced Neutrophil Mobilization *In Vivo*

**Figure 1:**
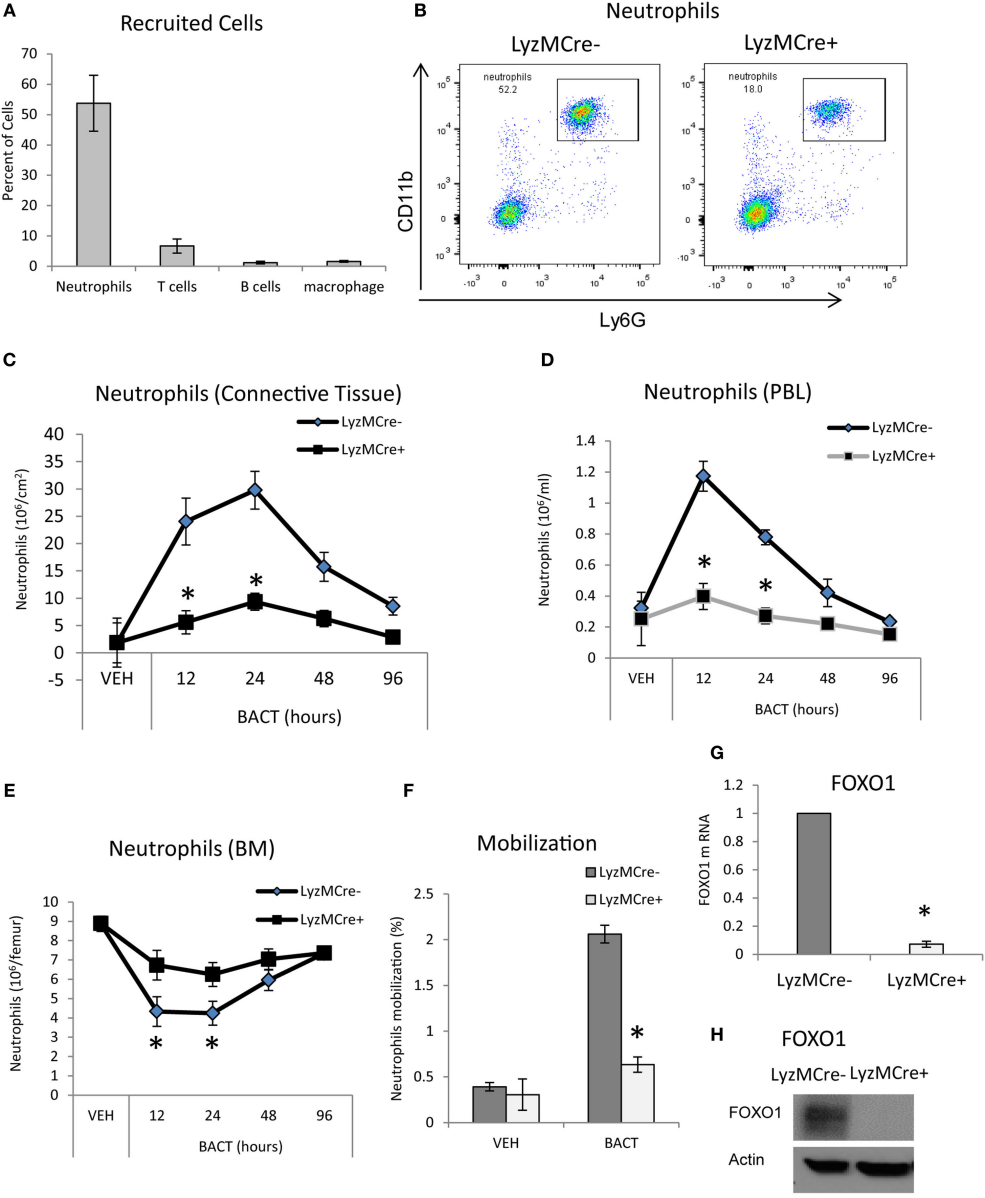
FOXO1 deletion impairs bacteria-induced neutrophil recruitment. **(A,B)** *Porphyromonas gingivalis* was inoculated into the connective tissue of the scalp and mice were examined at the indicated time points. **(A)** The mice was euthanized at 12 h and number of cells in the inoculated tissue and **(B)** neutrophils in peripheral blood were measured by immunofluorescent flow cytometry using specific antibodies for neutrophils (Ly6G), T cells (CD3), B cells (B220), and macrophages (F4/80). FOXO1-deleted LyzM.Cre^+^FOXO1^L/L^ mice and littermate control LyzM.Cre^−^FOXO1^L/L^ mice were inoculated with bacteria at the indicated time points. The number of neutrophils in the inoculated tissue **(C)**, peripheral blood **(D)**, and bone marrow **(E)** was measured by immunofluorescent flow cytometry using specific antibodies. **(F)** Neutrophil mobilization was calculated as described in methods to estimate the percentage of total neutrophils in the blood. RNA was isolated from mouse neutrophils from FOXO1-deleted LyzM.Cre^+^FOXO1^L/L^ mice and littermate control LyzM.Cre^−^FOXO1^L/L^ mice. FOXO1 mRNA levels were measured by RT-PCR and normalized to ribosomal protein L32 **(G)**. **(H)** Neutrophils from experimental LyzM.Cre^+^FOXO1^L/L^ mice and littermate control LyzM.Cre^−^FOXO1^L/L^ mice were analyzed by Western blots for FOXO1 expression with actin as loading control. The data are representative of two or three independent experiments. Data are presented as the mean ± SEM from triplicate samples. *Significant difference between neutrophils from FOXO1-deleted LyzM.Cre^+^FOXO1^L/L^ mice and littermate control LyzM.Cre^−^FOXO1^L/L^ mice (*P* < 0.05).

To examine host bacteria interactions *in vivo*, we utilized a well-defined animal model in which bacteria are inoculated into the scalp connective tissue (23–25). The number of mature neutrophils, T cells, B cells, and macrophages recruited to the site of inoculation following injection of bacteria was measured by immunofluorescent flow cytometry using specific antibodies or matched control antibody. As expected, the major leukocyte recruited were neutrophils, which were far greater than T cells, B cells, and macrophages (*P* < 0.05) (Figure 1A). To determine whether FOXO1 plays a role in bacteria-induced neutrophil recruitment, experimental mice were examined in which floxed FOXO1 was deleted in myeloid cells by Cre recombinase under the control of a LyzM promoter element (LyzM.Cre^+^FOXO1^L/L^) and compared with littermate controls (Figures 1B–F). The expression of CD11c.Cre^+^ recombinase has no apparent affect as demonstrated by comparison with wild-type mice as reported (35). Bacterial inoculation stimulated a 16-fold increase in the number of neutrophils recruited in WT control mice at 24 h, which was reduced 80% in LyzM.Cre^+^FOXO1^L/L^ experimental mice (*P* < 0.05) (Figure 1C). The number of mature neutrophils in the blood (Figures 1B,D) and BM (Figure 1E) was measured by immunofluorescent flow cytometry to examine the role of FOXO1 in neutrophil redistribution following inoculation of bacteria. After 12 h, there was a 4.6-fold increase in neutrophil numbers in the PBL of control mice, which was 68% lower in experimental LyzM.Cre^+^FOXO1^L/L^ mice (*P* < 0.05) (Figure 1D). This coincided with a 52% decrease in the number of neutrophils in the BM of control mice 12 h following bacterial inoculation. Thus, deletion of FOXO1 in LyzM.Cre^+^FOXO1^L/L^ experimental mice caused a significant reduction in the movement of neutrophils from the BM to PBL after bacterial inoculation (*P* < 0.05) (Figure 1E). Quantitatively, bacteria induced a fivefold increase in neutrophil mobilization from the BM compartment to the peripheral vasculature in WT control mice at 12 h, which was reduced by more than 50% in FOXO1-deleted experimental mice *in vivo* (Figure 1F). To ensure that FOXO1 was deleted, neutrophils from LyzM.Cre^+^FOXO1^L/L^ and matched control mice were examined (*P* < 0.05) (Figures 1G,H). At both the mRNA and protein level, the results demonstrate efficient FOXO1 deletion in neutrophils in the experimental but not control groups.

### FOXO1 Deletion Interferes with Neutrophil Chemotaxis

**Figure 2:**
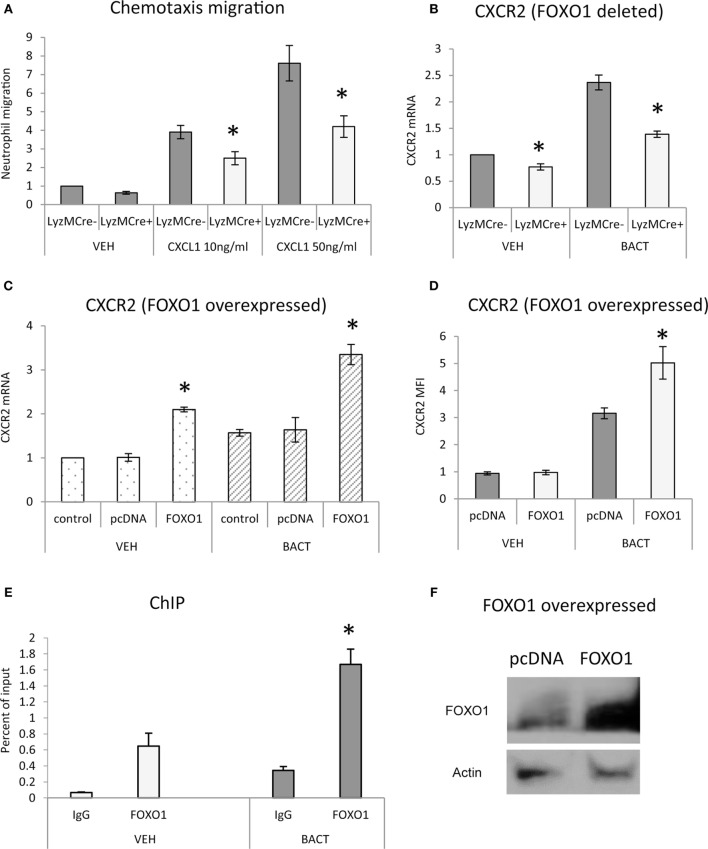
FOXO1 is needed for neutrophil migration and CXCR2 expression. **(A)** Migration was examined in neutrophils from FOXO1-deleted LyzM.Cre^+^FOXO1^L/L^ mice or littermate control LyzM.Cre^−^FOXO1^L/L^ mice in transwell chambers. CXCL1 was added to the bottom chamber and neutrophils that migrated to the bottom chamber were quantified following DAPI staining and fluorescence microscopy. **(B)** Bacteria were incubated with neutrophils from FOXO1-deleted LyzM.Cre^+^FOXO1^L/L^ and control LyzM.Cre^−^FOXO1^L/L^ mice. CXCR2 mRNA levels were measured by RT-PCR and normalized to ribosomal protein L32. **(C)** HL-60 neutrophils were transfected with FOXO1 or empty vector alone and stimulated with bacteria or vehicle alone. RNA was isolated from neutrophils. CXCR2 expression was measured by real-time PCR. **(D)** CXCR2 protein levels were assessed by mean fluorescence intensity (MFI). **(E)** Chromatin immunoprecipitation (ChIP) assays were performed with neutrophils from control LyzM.Cre^−^FOXO1^L/L^ mice. **(F)** HL-60 neutrophils were transfected with FOXO1 expression plasmid or empty vector alone and FOXO1 protein levels were measured by immunoblot with a specific antibody. Actin was assessed as a loading control. The data are representative of two or three independent experiments. Data are expressed as the mean ± SEM from triplicate samples. *Significant difference between neutrophils from FOXO1-deleted LyzM.Cre^+^FOXO1^L/L^ mice and littermate control LyzM.Cre^−^FOXO1^L/L^ mice (*P* < 0.05). *Significant difference between HL-60 neutrophils from transfection of FOXO1 vs. empty vector alone (*P* < 0.05).

To determine whether FOXO1 facilitates neutrophil migration, *in vitro* studies were carried out. Neutrophils were stimulated with the chemokine CXCL1 and examined in a transwell assay. CXCL1 induced a dose-dependent increase in migration that was stimulated up to sevenfold in control mice and reduced by 45% when FOXO1 was deleted (*P* < 0.05) (Figure 2A). To examine how FOXO1 may affect neutrophil migration, CXCR2, a receptor for CXCL1, was assessed. Bacteria induced a 2.3-fold increase in CXCR2 mRNA levels (*P* < 0.05) that was 44% lower in similarly stimulated neutrophils from experimental littermates (*P* < 0.05) (Figure 2B). Regulation of CXCR2 by FOXO1 was further examined by transfection of HL-60 neutrophils with a FOXO1 expression plasmid. FOXO1 plasmid increased CXCR2 mRNA levels 2.2-fold and protein levels by 1.6-fold compared to empty vector (Figures 2C,D). Moreover, the increase was further enhanced in cells stimulated with bacteria, suggesting that FOXO1 enhances CXCR2 in cooperation with other factors that are induced in neutrophils stimulated with bacteria. Direct interaction between FOXO1 and the CXCR2 promoter was demonstrated by ChIP assay, which was also significantly enhanced in bacteria stimulated neutrophils (*P* < 0.05) (Figure 2E). Furthermore, FOXO1 protein levels were increased in neutrophils transfected with a FOXO1 expression plasmid (*P* < 0.05) (Figure 2F). Thus, FOXO1 mediates CXCR2 transcription stimulated by bacteria and deletion of FOXO1 in neutrophils reduces chemotaxis induced by its cognate ligand.

### FOXO1 Deletion Impairs Neutrophil Phagocytosis and Bacterial Killing

**Figure 3:**
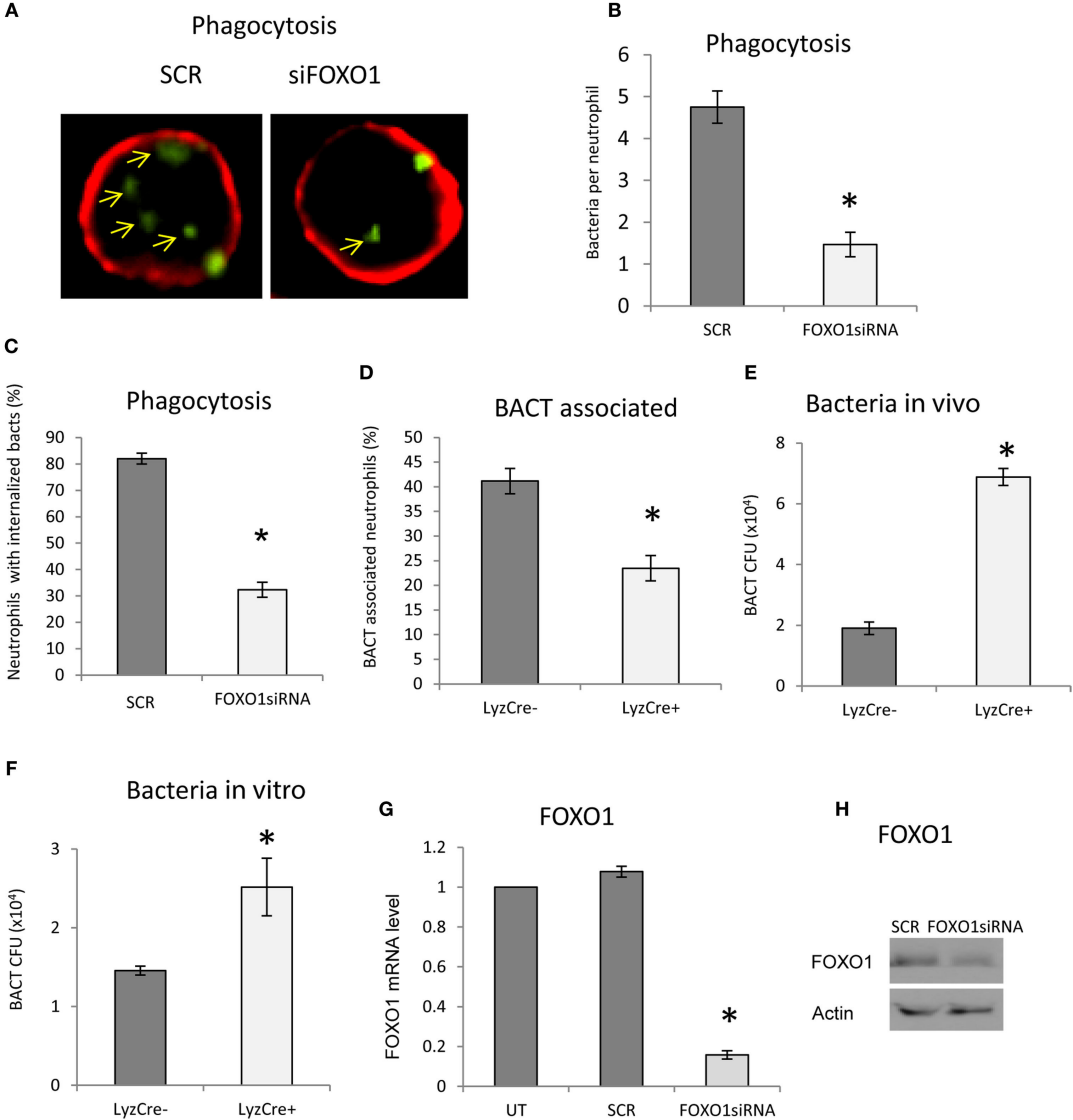
FOXO1 deletion impairs neutrophil bacterial phagocytosis. **(A)** Carboxyfluorescein succinimidyl ester (*CFSE*)-labeled bacteria were incubated with human HL-60 neutrophils transfected with scrambled or FOXO1siRNA. Deconvolution fluorescent microscopic images were taken after the neutrophils were stained with Alexa 647-labeled wheat germ agglutinin (WGA) to delineate the cell surface (red) and internalized bacteria (green). Internalized bacteria were considered as those within WGA-labeled plasma membranes. **(B)** Internalized bacteria expressed as the number of bacteria internalized per cell. **(C)** The percent neutrophils with phagocytosed bacteria. **(D)** The percent neutrophils with associated bacteria. **(E)** FOXO1-deleted LyzM.Cre^+^FOXO1^L/L^ and control LyzM.Cre^−^FOXO1^L/L^ mice were inoculated with *Porphyromonas gingivalis in vivo*. Live bacterial CFUs were measured. **(F)** Neutrophils from FOXO1-deleted LyzM.Cre^+^FOXO1^L/L^ mice and littermate control LyzM.Cre^−^FOXO1^L/L^ mice were isolated and incubated with *P*. *gingivalis in vitro*. Viable bacterial CFUs were counted. **(G)** RNA was isolated from neutrophils and FOXO1 was measured by real-time PCR. **(H)** Neutrophils transfected with the siRNA were analyzed by Western blots using a antibody specific for FOXO1. Actin was assessed as a loading control. The data are representative of two or three independent experiments of mean ± SEM from triplicate samples. *Significant difference between human neutrophils from transfection of FOXO1 siRNA vs. scramble siRNA (*P* < 0.05). *Significant difference between neutrophils from FOXO1-deleted LyzM.Cre^+^FOXO1^L/L^ mice and littermate control LyzM.Cre^−^FOXO1^L/L^ mice (*P* < 0.05). SCR, scrambled siRNA; UT, untransfected control.

The impact of FOXO1 deletion on neutrophil phagocytosis was examined. Phagocytosis was assessed by internalization of labeled bacteria. Neutrophils phagocytosis of bacteria was reduced ~60% (Figures 3A–C) (*P* < 0.05) when FOXO1 was knocked down. The number of neutrophils with associated bacteria was reduced in half (*P* < 0.05) (Figure 3D). Since a primary function of neutrophils is bacterial killing, we assessed this parameter *in vivo* 12 h after injection of bacteria and *in vitro* 2 h after coincubation, time points at which neutrophils are the primary antibacterial defense (36). *In vivo*, FOXO1 deficient mice were 70% less efficient in clearing bacteria than matched littermate control mice (*P* < 0.05) (Figure 3E). Similar results were obtained *in vitro*. Neutrophils from experimental mice with lineage specific FOXO1 deletion were 50% less efficient than control littermates in bacterial killing (*P* < 0.05) (Figure 3F). Experiments were carried out to ensure that FOXO1 was efficiently knocked down by RNAi. At both the mRNA (Figure 3G) and protein levels (Figure 3H), FOXO1 siRNA substantially reduced FOXO1 compared to scramble siRNA.

### Bacteria Induce FOXO1 Nuclear Localization

**Figure 4:**
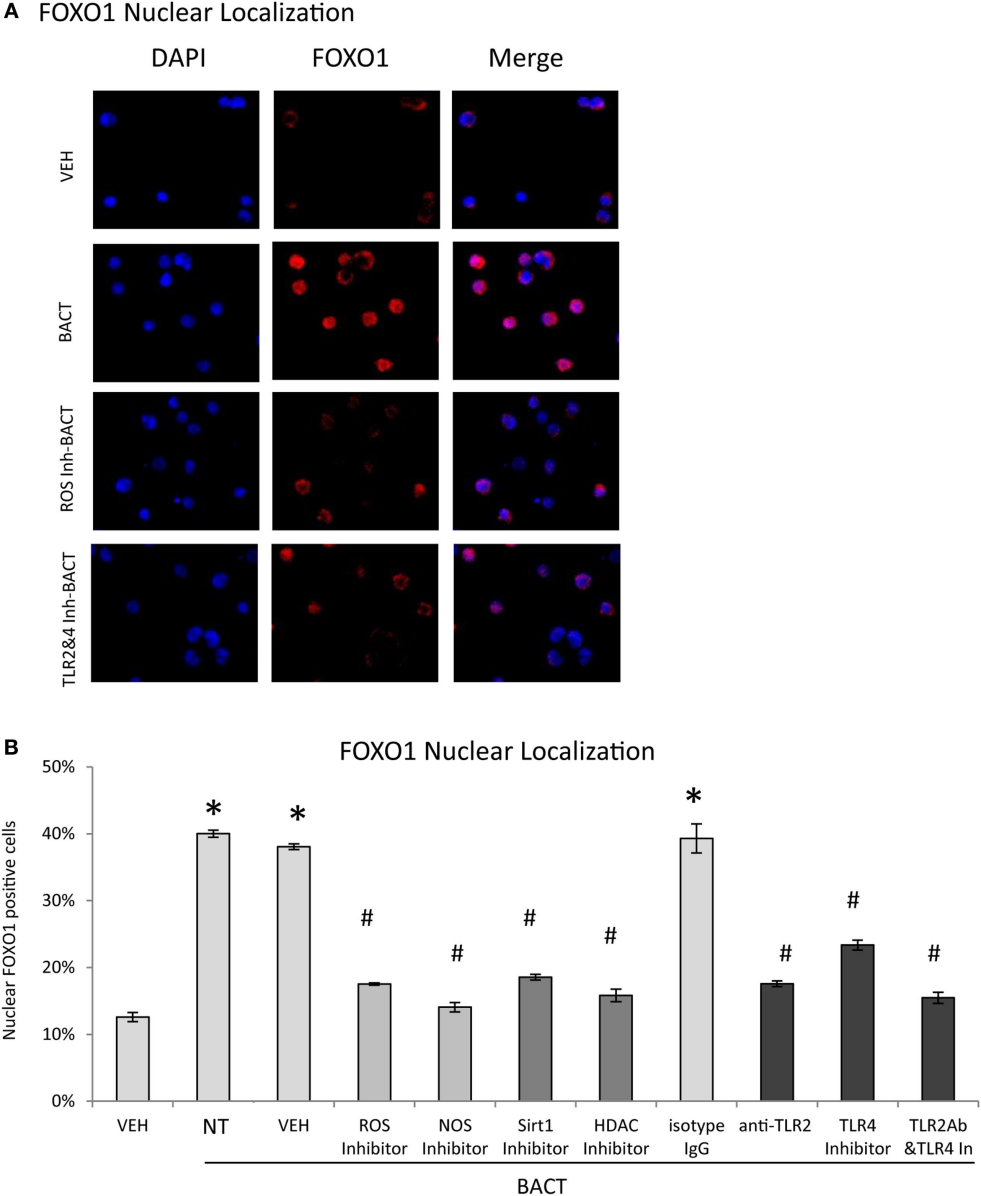
Bacteria stimulate FOXO1 nuclear localization through TLR signaling. **(A)** HL-60 neutrophils were incubated with TLR2 blocking antibody or matched control IgG or TLR4 inhibitor (TAK242) individually or combined. Cells were alternatively incubated with inhibitors to reactive oxygen species (ROS, NAC), NOS (L-NAME), Sirt1(Sirtinol), histone deacetylases (HDAC, Trichostatin A, TSA), or control vehicle alone, DMSO. **(A)** FOXO1 nuclear localization was determined by immunofluorescence using an antibody specific to FOXO1 and colocalization with DAPI nuclear stain. **(B)** Quantitation was determined by percent neutrophils with FOXO1 nuclear translocation. The data are representative of two or three independent experiments and expressed as the mean ± SEM from triplicate samples. *Significant difference between neutrophils incubated with bacteria or phosphate-buffered saline (*P* < 0.05) or ^#^treated with inhibitor and vehicle or isotype IgG control (*P* < 0.05).

Experiments were undertaken to better understand mechanisms through which bacteria stimulate FOXO1 nuclear localization, a key step in induction of FOXO1 activity. Bacteria stimulated FOXO1 nuclear localization by approximately fourfold. Bacteria-stimulated FOXO1 nuclear localization was dependent upon TLRs since inhibitors of TLR2 and TLR4 blocked most of this increase (*P* < 0.05) (Figures 4A,B). Inhibition of ROS and NOS, intermediates in TLR signaling also substantially reduced bacteria-induced FOXO1 nuclear localization. Furthermore, deacetylation of FOXO1 played an important role as inhibitors that blocked FOXO1 deacetylation, Sirt1 and HDAC largely blocked the ability of bacteria to stimulate translocation of FOXO1 to the nucleus (*P* < 0.05) (Figures 4A,B).

### FOXO1 Regulates CD11b Expression

**Figure 5:**
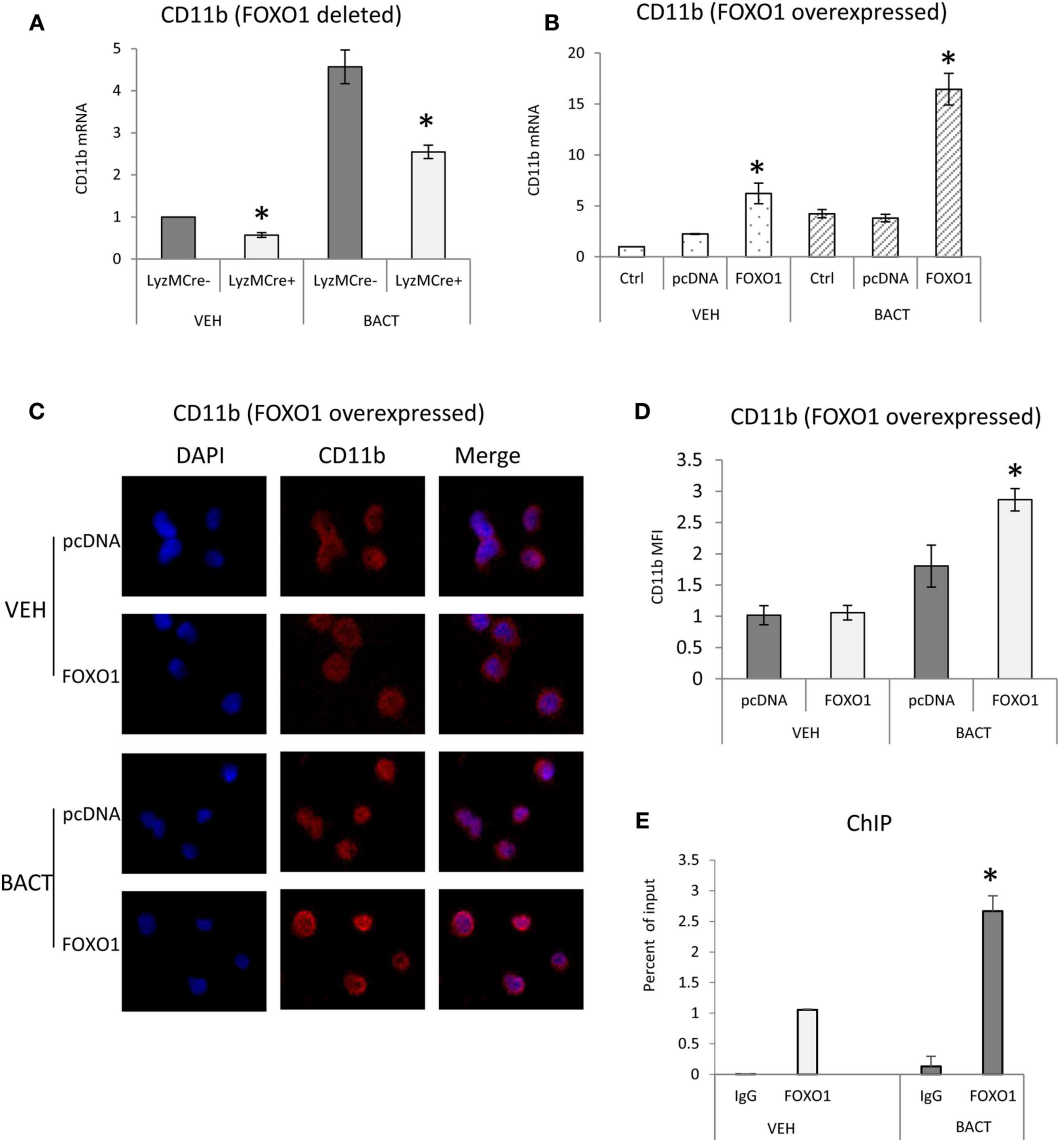
FOXO1 regulates CD11b expression. **(A)** Neutrophils were isolated from FOXO1-deleted LyzM.Cre^+^FOXO1^L/L^ and control LyzM.Cre^−^FOXO1^L/L^ mice and incubated with *Porphyromonas gingivalis*. **(A)** RNA was isolated from neutrophils and CD11b mRNA levels were measured by RT-PCR and normalized to ribosomal protein L32. **(B)** HL-60 neutrophils were transfected with a plasmid expressing FOXO1 or empty vector alone with or without incubation with bacteria. RNA was isolated from neutrophils and CD11b measured by real-time PCR. **(C)** HL-60 neutrophils were transfected with FOXO1 expression plasmic or vector alone. Immunofluorescence was carried out using a CD11b specific antibody and DAPI counterstain. Intensity was measured to assess protein levels of CD11b. **(D)** Cells described in panel C were assessed for mean fluorescence intensity (MFI) to quantify CD11b protein levels. **(E)** FOXO1 interaction with the CD11b promoter examined by chromatin immunoprecipitation (ChIP) assay using primary murine neutrophils from normal mice. The data are representative of two or three independent experiments and expressed as the mean ± SEM from triplicate samples. *Significant difference between neutrophils from FOXO1-deleted LyzM.Cre^+^FOXO1^L/L^ mice and littermate control LyzM.Cre^−^FOXO1^L/L^ mice (*P* < 0.05). *Significant difference between neutrophils from transfection of FOXO1 vs. empty vector alone (*P* < 0.05).

To investigate a potential mechanism by which FOXO1 affects several aspects of neutrophil function including migration and phagocytosis (37), we examined FOXO1 regulation of CD11b. Under basal conditions neutrophils from mice with FOXO1 deletion had 50% less CD11b mRNA than control littermates (*P* < 0.05) (Figure 5A). Bacterial stimulation increased neutrophil CD11b mRNA levels almost fivefold *in vitro* (*P* < 0.05). More than 50% of this increase was blocked in neutrophils from experimental FOXO1-deleted mice (*P* < 0.05) (Figure 5A). To further investigate regulation of CD11b, HL-60 neutrophils were transfected with FOXO1 or empty vector alone and stimulated with bacteria. Overexpression of FOXO1 induced a significant 3.9-fold increase in CD11b mRNA levels and 1.6-fold increase at the protein level compared to empty vector (Figures 5B–D). To determine whether FOXO1 regulates neutrophil activity directly, interaction between FOXO1 and the CD11b promoter was examined by ChIP assay. Under basal conditions FOXO1 was shown to interact directly with the CD11b promoter, which was increased almost threefold in neutrophils stimulated by bacteria (*P* < 0.05) (Figure 5E).

### FOXO1 Regulates Bacteria-Induced TLR and Cytokines Expression

**Figure 6:**
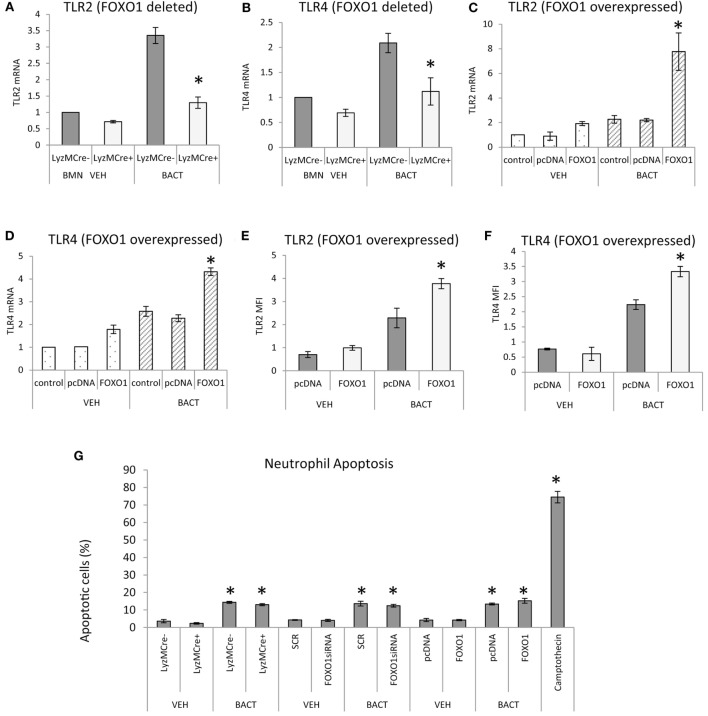
FOXO1 regulates TLR expression in neutrophils. **(A,B)** Bacteria were incubated with neutrophils from FOXO1-deleted LyzM.Cre^+^FOXO1^L/L^ and littermate control LyzM.Cre^−^FOXO1^L/L^ mice. RNA was isolated and mRNA levels of TLR2 and TLR4 were measured by RT-PCR and normalized to ribosomal protein L32. **(C–F)** HL-60 neutrophils were transfected with FOXO1 or empty vector alone and stimulated with bacteria or vehicle alone. RNA was isolated and real-time PCR was carried out to assess mRNA levels of TLR2 and TLR4 or immunofluorescence was carried out with antibody specific for TLR2 or TLR4 and protein levels were assessed by mean fluorescence intensity (MFI). **(G)** Neutrophils were isolated from bone marrow of FOXO1-deleted LyzM.Cre^+^FOXO1^L/L^ mice and littermate control LyzM.Cre^−^FOXO1^L/L^ mice by break free centrifugation on Histopaque 1119 and 1077 and washed by phosphate-buffered saline (PBS) without calcium/magnesium. Alternatively HL-60 neutrophils were transfected with scrambled siRNA (SCR) or FOXO1 siRNA or were transfected with FOXO1 expression plasmid (FOXO1) or empty vector alone (pcDNA). Cells were incubated with *P. gingivalis* (Bact) (multiplicity of infection 1:10) for 12 h or vehicle alone (VEH) at 37°C with 5% CO_2_ at normoxic moisture conditions. Apoptotic cells were assessed by flow cytometry with Annexin V labeling. The positive control was HL-60 neutrophils incubated with camptothecin. The data are representative of two or three independent experiments and expressed as the mean ± SEM from triplicate samples. *Significant difference between neutrophils from FOXO1-deleted LyzM.Cre^+^FOXO1^L/L^ mice and littermate control LyzM.Cre^−^FOXO1^L/L^ mice (*P* < 0.05). *Significant difference between HL-60 neutrophils from transfection of FOXO1 vs. empty vector alone or human neutrophils scrambled or FOXO1siRNA transfected (*P* < 0.05).

Bacterial stimulation increased TLR2 and TLR4 mRNA levels twofold to threefold and most of this increase was blocked in neutrophils with deleted FOXO1 (*P* < 0.05) (Figures 6A,B). Similarly, overexpression of FOXO1 increased TLR2 and TLR4 mRNA and protein levels particularly in neutrophils coincubated with bacteria (Figures 6C–F). Thus, FOXO1 can potentially sensitize neutrophils to bacterial stimulation through upregulation of TLRs to enhance inflammatory responses. To determine whether changes in neutrophils modulated by FOXO1 could be due to apoptosis, experiments were carried out assessing apoptosis by Annexin V. Bacteria stimulated a small increase in neutrophil apoptosis that was much less than the positive control, camptothecin (Figure 6G). However, deletion of FOXO1 by Cre recombinase or knockdown of FOXO1 by siRNA had no effect on neutrophil apoptosis.

**Figure 7:**
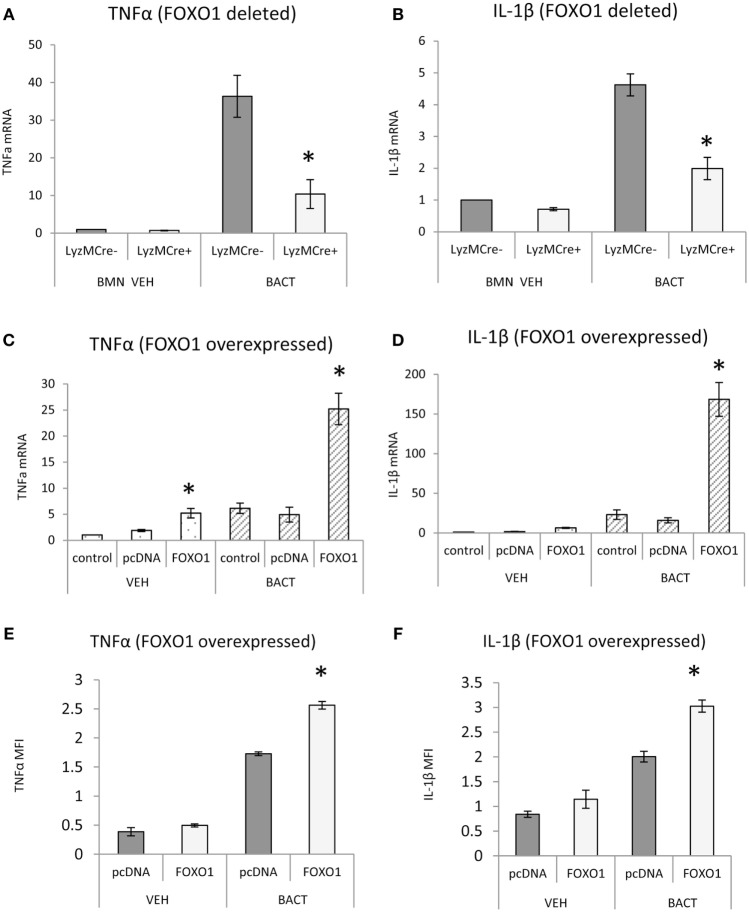
FOXO1 regulates TNFα and IL-1β. **(A,B)** Neutrophils from FOXO1-deleted LyzM.Cre^+^FOXO1^L/L^ mice and littermate control LyzM.Cre^−^FOXO1^L/L^ mice were incubated with *Porphyromonas gingivalis*. RNA was isolated from neutrophils and mRNA levels of TNFα or IL-1β were measured by RT-PCR and normalized to ribosomal protein L32. **(C–F)** HL-60 neutrophils were transfected with FOXO1 or empty vector alone and stimulated with bacteria or vehicle. **(C,D)** RNA was isolated from neutrophils and mRNA levels of TNFα and IL-1β measured by real-time PCR. **(E,F)** Cells were incubated with anti-TNFα or anti-IL-1β antibody (sc-7884, Rabbit Polyclonal IgG 1:50, Santa Cruz Biotechnology, Santa Cruz, CA, USA) overnight and then with secondary antibody, donkey antirabbit biotinylated antibody (1:200, Jackson ImmunoResearch Laboratories, West Grove, PA) for 1 h at room temperature in a box with the humidified atmosphere, followed by incubation with streptoavidin-Alexa 546 (S-11225, 1:400, for 1 h, Thermo Fisher Scientific). The mean fluorescence intensity (MFI) was measured to assess protein levels of each. The data are representative of two or three independent experiments and expressed as the mean ± SEM from triplicate samples. *Significant difference between neutrophils from FOXO1-deleted LyzM.Cre^+^FOXO1^L/L^ mice and littermate control LyzM.Cre^−^FOXO1^L/L^ mice (*P* < 0.05). *Significant difference between HL-60 neutrophils transfected with FOXO1 expression plasmid vs. empty vector alone for mRNA or protein level (*P* < 0.05).

FOXO1 may contribute to inflammatory responses of neutrophils by upregulating cytokine expression. FOXO1 deletion in neutrophils reduced the capacity of bacteria to induce TNFα and IL-1β mRNA levels by 50–70% (*P* < 0.05) (Figures 7A,B). Although IL-1β mRNA levels are not directly related to mature IL-1β protein levels, the results do show FOXO1 regulation IL-1β mRNA. The effect of transfection of a FOXO1 expression plasmid on TNFα and IL-1β was also assessed. Transfection with FOXO1 stimulated approximately fivefold increase in TNFα and IL-1β mRNA (Figures 7C,D). When neutrophils were stimulated with bacteria plus FOXO1 overexpression the levels were further increased to 25-fold for TNFα and >100-fold for IL-1β. A similar synergy between FOXO1 overexpression and bacterial stimulation was observed when the protein levels were measured by MFI (Figures 7E,F).

## Discussion

FOXO1 is a transcription factor present in many cell types. Previous studies demonstrated that FOXO1 regulates cytokine production in dendritic cells and macrophages (38), dendritic cell homing to lymph nodes and lymphocyte activation (16, 18, 39) Lymphocyte trafficking to secondary lymphoid organs (40) and formation of germinal centers (18, 41–43) is also FOXO1 dependent. We show here for the first time that FOXO1 is needed to mobilize neutrophils from the BM to the vasculature and to recruit neutrophil to sites of bacterial inoculation. Moreover, FOXO1 plays a critical role in upregulating antibacterial neutrophil responses that clear bacterial infection including phagocytosis and bacterial killing.

Mobilization of neutrophils from the BM to the vasculature is an early and important step in the response to bacterial infection (44). The BM is the site of neutrophil production where neutrophils mature and are released into the circulation. Infection causes a relocation of neutrophils by mobilizing their release from BM followed by an increase in circulating neutrophils and recruitment to the infected site (44). FOXO1 deletion in neutrophils significantly reduced neutrophil mobilization from the BM. This was reflected in both an increase in the number of neutrophils that were present in the BM of experimental mice and the reduced numbers that were circulating following inoculation of bacteria. CXCR2 is critical for neutrophil mobilization as neutrophils lacking CXCR2 are retained in the BM and have a reduced mobilization from BM to vasculature (5). The role of CXCR2 is based on the findings that recruitment of neutrophils to the lungs following *Streptococcus pneumoniae* infection is reduced by CXCR2 ablation (45). Since CXCR2 is the primary chemokine receptor that regulates neutrophil mobilization, we determined whether it was regulated by FOXO1. FOXO1 interacted directly with the CXCR2 promoter, FOXO1 deletion reduced bacteria-induced CXCR2 *in vitro* and *in vivo* and FOXO1 overexpression increased its mRNA levels. These results strongly support the capacity of FOXO1 to regulate expression of CXCR2 and thereby modulate neutrophil mobilization. However, it does not rule out the possibility that FOXO1 deletion in macrophages also affects neutrophil recruitment in the experimental mice. It is noteworthy that the number of neutrophils in the BM did not change with FOXO1 deletion under steady-state conditions indicating that the changes that we observed were due to mobilization of neutrophils rather than their production or maturation.

We found that deletion of FOXO1 significantly reduced the clearance of inoculated bacteria. This was shown by a fourfold reduction in bacteria present in matched control mice compared to mice with FOXO1 deletion. Most of this reduction occurred within a 16 h time frame in which the majority of bacteria removed by the innate immune response is due to the activity of neutrophils (36). Within the time frame of the study by far the predominant leukocyte was neutrophils, with small amounts of T cells followed by macrophages and B cells, consistent with other studies that neutrophils are predominantly responsible for early clearance of bacteria following infection (27). Ablation of FOXO1 in neutrophils significantly reduced their capacity to phagocytize bacteria and kill bacteria *in vitro*. The former is likely due to FOXO1 regulation of CD11b, which interacts with CD18 to play an important role in capture of bacteria (46). CD11b promotes phagocytosis of bacteria (47). FOXO1 ablation also reduced the number of neutrophils recruited to the site of bacterial inoculation. The reduced numbers of neutrophils may also negatively impact the ability to clear bacteria *in vivo*. In addition to *P. gingivalis*, we have also examined FOXO1 dependent neutrophil responses to a Gram-positive bacterium, *Bacillus subtilis*. *B. subtilis* stimulated FOXO1 nuclear localization; neutrophil phagocytosis and killing of *B. subtilis in vitro* was FOXO1 dependent (data not shown).

Bacteria induce FOXO1 activation through TLR2/4 as shown by significantly reduced FOXO1 nuclear localization with TLR2/4 inhibitors. This is likely to be mediated by ROS/NOS and deacetylation since bacteria-induced FOXO1 nuclear localization in neutrophils was reduced by inhibition of ROS/NOS and deacetylation inhibitors including an inhibitor of SIRT1. This is consistent with findings that FOXO1 nuclear localization is stimulated by induction of ROS/NOS (48) and TLR stimulates ROS and NOS production (49, 50). Similarly FOXO1 activation has been shown to be dependent upon its deacetylation (51). These results indicate that FOXO1 may sensitize neutrophils to bacterial stimulation through upregulation of TLR2/4 and enhance neutrophil-mediated inflammation by increasing inflammatory cytokine expression. The ability of FOXO1 to enhance inflammation in neutrophils is dependent upon generation of ROS/NOS and the deacetylation of FOX1. Furthermore, FOXO1 appears to positively interact with TLR signaling pathways as the upregulation of TNFα and IL-1β was much greater when neutrophils were transfected with a vector expressing FOXO1 and stimulated with bacteria compared to bacterial stimulation or FOXO1 transfection alone.

In summary, we describe a novel function for FOXO1 in regulating neutrophil activity *in vivo*, particularly chemotaxis, stimulation of bacterial phagocytosis, and bacterial clearance. Moreover, bacteria-induced activation of FOXO1 was dependent upon TLR2 and/or TLR4 and FOXO1 overexpression significantly enhanced cytokine expression induced by bacterial stimulation. FOXO1 regulated several downstream genes that affect neutrophil function including CXCR2 and CD11b which play an important role in neutrophil response to bacterial challenge. Thus FOXO1 coordinates upregulation of neutrophil activity through key downstream target genes to modulate neutrophil function.

## Ethics Statement

This study was carried out in accordance with the recommendations of the guidelines of the University of Pennsylvania Institutional Animal Care and Use Committee. The protocol was approved by University of Pennsylvania Institutional Animal Care and Use Committee.

## Author Contributions

GD and DG conceived and designed the research. GD, LS, CT, YW, FM, JZ, CL, and SA performed experiments. GD and DG analyzed the data. GD and DG interpreted the results. GD and DG prepared figures and drafted the manuscript. GD, LS, and DG edited and revised the manuscript. All authors approved the final manuscript version.

## Conflict of Interest Statement

The authors declare that the research was conducted in the absence of any commercial or financial relationships that could be construed as a potential conflict of interest.

## Abbreviations

ROS, reactive oxygen species; NOS, nitric oxide synthase; Sirt1, NAD-dependent deacetylase sirtuin-1; HDAC, histone deacetylases; TLR, toll-like receptor; *CFSE*, carboxyfluorescein succinimidyl ester; WGA, wheat germ agglutinin; MOI, multiplicity of infection; MFI, mean fluorescence intensity; Inh, inhibitor; PBL, peripheral blood; BM, bone marrow.
